# Every Breath You Take: The Impact of Environment on Resident Memory CD8 T Cells in the Lung

**DOI:** 10.3389/fimmu.2014.00320

**Published:** 2014-07-08

**Authors:** Hillary L. Shane, Kimberly D. Klonowski

**Affiliations:** ^1^Department of Cellular Biology, University of Georgia, Athens, GA, USA

**Keywords:** CD8+ T cells, memory T cells, tissue-resident memory cells, influenza A virus, lung

## Abstract

Resident memory T cells (T_RM_) are broadly defined as a population of T cells, which persist in non-lymphoid sites long-term, do not re-enter the circulation, and are distinct from central memory T cells (T_CM_) and circulating effector memory T cells (T_EM_). Recent studies have described populations of T_RM_ cells in the skin, gut, lungs, and nervous tissue. However, it is becoming increasingly clear that the specific environment in which the T_RM_ reside can further refine their phenotypical and functional properties. Here, we focus on the T_RM_ cells that develop following respiratory infection and reside in the lungs and the lung airways. Specifically, we will review recent studies that have described some of the requirements for establishment of T_RM_ cells in these tissues, and the defining characteristics of T_RM_ in the lungs and lung airways. With continual bombardment of the respiratory tract by both pathogenic and environmental antigens, dynamic fluctuations in the local milieu including homeostatic resources and niche restrictions can impact T_RM_ longevity. Beyond a comprehensive characterization of lung T_RM_ cells, special attention will be placed on studies, which have defined how the microenvironment of the lung influences memory T cell survival at this site. As memory T cell populations in the lung airways are requisite for protection yet wane numerically over time, developing a comprehensive picture of factors which may influence T_RM_ development and persistence at these sites is important for improving T cell-based vaccine design.

## Introduction

The adaptive immune system is defined by its ability to mount an antigen-specific immune response and generate long-lived memory cells. CD8^+^ memory T cells (T_mem_) respond rapidly upon secondary encounter with the same antigen and can provide protection against the development of severe disease or chronic infection in the absence of neutralizing antibodies (1). This attribute of T_mem_ is particularly attractive in the context of vaccine design for viral infections such as HIV or influenza, which rapidly modify antibody targets as a result of high mutagenic rates and immune pressure.

The efficiency of T_mem_-mediated protection is in part a direct result of activated T cells initiating divergent developmental and migratory programs, which provide the host with a multifaceted immune response following challenge. This T_mem_ diversity is acquired as a result of different levels of co-stimulation, inflammation, or T cell help, which not only vary throughout the course of a single infection but are also impacted by infection route. Initially, memory T cells were broadly categorized into two populations based on homing preferences, circulating between secondary lymphoid organs as central memory T cells (T_CM_) or less discretely throughout the periphery, including non-lymphoid tissues, defined as effector memory T cells (T_EM_) (2). These memory pools are distinguished from one another by their differential expression of the lymph node homing molecules L-selectin (CD62L) and CCR7, with T_CM_ expressing high levels of these molecules for lymph node entry and retention (3) and T_EM_ cells expressing low levels. While this simplified T_CM_/T_EM_ paradigm predominated T_mem_ classification for several years, subsequent studies using parabiotic mice (4) and adoptive transfer systems (5) demonstrated that at least one additional T_mem_ pool exists with tissue-specific residency and little migratory potential. Additional studies confirmed the existence of these tissue-locked T_mem_ at portals of pathogen entry and led to the T resident memory cells (T_RM_) nomenclature.

As relative newcomers to the T cell memory scene, T_RM_ cells have not been characterized to the same extent as T_CM_ and T_EM_ cells, and our definition of this memory population, as well our understanding of its origin is still evolving. Nonetheless, specific CD8^+^ T_RM_ populations have been identified in many peripheral sites including the gut (6), skin (7), brain (8), female reproductive mucosa (9, 10), and the lung (11). Despite some similarities with T_EM_, lack of equilibration of T_mem_ between specific tissues of parabiotic mice as well as general “hallmarks” of T_RM_ have been identified as defining characteristics. These distinguishing features include the expression of CD103 (α_E_ integrin) and CD69, molecules traditionally associated with adhesion within epithelial layers and recent activation, respectively (12, 13). A recent paper by Mackay et al. defined a common transcriptional signature shared by CD103^+^ T_RM_ cells isolated from the skin, gut, and lung consisting of 37 genes differentially expressed compared to T_EM_ or T_CM_ cells, demonstrating that T_RM_ cells are a distinct T_mem_ lineage (14). Additionally, this study determined that T_RM_ cells from distinct anatomical sites also possessed unique gene transcription patterns, with 127 being unique to the gut, 86 unique to the skin, and 25 unique to the lung, indicating additional diversification within the T_RM_ pool, likely environmentally driven.

Despite the relative juvenescence of the T_RM_ field, the importance of this cell population has been alluded to for some time. T_RM_ cells are positioned at the site of pathogen encounter as a front line of defense, and several studies have highlighted their role in defense against pathogenic challenges (7, 15–17). Indeed, in the case of influenza virus infection, the number of antigen-specific CD8^+^ T cells located within the respiratory tract correlates with the highest degree of heterosubtypic immunity (18, 19), and recently it has been shown that T_RM_ specifically are responsible for this protection (20). Defining the characteristics that lead to T_RM_ development, and determining how they persist at sites of infection may lead to novel ways to enhance vaccine efficacy. This review will focus on the development, characteristics, and maintenance of CD8^+^ T_RM_ cells in the respiratory tract, which develop after acute respiratory infection, primarily with influenza and Sendai viruses. How the lung environment affects the developmental transition and tissue residency of CD8^+^ T_RM_ cells will be discussed, from the primary activation of the antigen-specific cell through the return to homeostasis and during resting conditions.

## Part I: Factors Influencing T_RM_ Development

**Figure 1:**
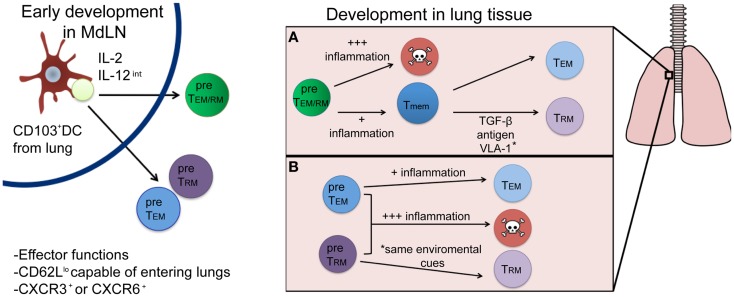
**Proposed developmental pathways for T_RM_ cells following respiratory infection**. Priming by a CD103^+^ DC and appropriate cytokine signals (left) results in the generation of either a common T_EM/RM_ precursor cell (green) or individual pre-T_EM_ or T_RM_ cells (blue and purple, respectively). Once in the lung (right), environmental factors will drive subsequent cell fate decisions, resulting in either terminal differentiation (and death) or the generation of memory cells. While most current evidence supports the differentiation route depicted in **(A)**, where a common precursor differentiates first into memory, followed by environmentally driven lineage differentiation into T_RM_ or T_EM_ cells, it is plausible that differentiation into a T_RM_ fate occurs immediately following priming in the lymph node **(B)** and is distinguished by yet to be discovered phenotypic or genetic markers.

There is great interest in deciphering the T_RM_ developmental pathway, as understanding this mechanism could lead to modulation of the responses in ways, which could enhance the establishment of this T_mem_ pool. The development of T_RM_ cells will have two main requirements: (1) the ability to survive through contraction (become a T_mem_) and (2) the ability to differentiate into the appropriate memory lineage (become a T_RM_ cell as opposed to a T_EM_ or T_CM_ cell). In this section, we will discuss factors that may influence T_RM_ development in the early priming environment of the lymph node, and subsequently in the inflamed lung. Recent evidence demonstrates that T cell differentiation into distinct T_EM_ and T_CM_ subsets occurs soon after T cell priming (21), which begs the question: does a population of cells that is destined to become T_RM_ cells also develop during or soon after initial activation in the lymph node? Or, do T_RM_ arise only after tissue-specific entry based on specific cues within the microenvironment of tissues, like the lung? These scenarios are not necessarily mutually exclusive and full commitment to the T_RM_ lineage is likely due to a combination of these two possibilities as will be further discussed and as described in Figure 1.

### Early differentiation signals in the lymph node: Developing T_RM_ potential

Activation of CD8^+^ T cell requires three signals: detection of cognate peptide/MHCI complex, co-stimulation, and a cytokine signal (22). The combination of these three signals, which may vary in intensity and type, results not only in clonal expansion and acquisition of effector function, but also influences long-term cellular fate (21). In many cases, the overall T_mem_ potential of the antigen-specific CD8^+^ T cell is driven by lineage-associated transcription factors and acquired epigenetic changes (23), which can be experimentally monitored. These programing signals are influenced by the type of (priming) APC, antigen availability, and inflammatory properties of the pathogen, which can vary based on the individual pathogen and the route, which infection is acquired. While an early T_RM_ lineage-specific transcriptional program has not been identified, specific migratory signals facilitating peripheral tissue entry, with subsequent acquisition of T_RM_ characteristics implies that at least some early signals help polarize cells toward a T_RM_ fate. Here, we will discuss the possible early signals encountered in the lung draining mediastinal lymph nodes (MdLN), which may promote the development of respiratory T_RM_ cells.

During influenza infection, activated, antigen-laden respiratory DCs migrate to the MdLN to interact with naïve CD8^+^ T cells. The majority of these migratory DCs fall into two subsets, airway localized CD103^+^ DCs and lung parenchyma CDllb^hi^ DCs (24). In addition to their localization in the lung during a resting state, these DC populations differ in their induction of CD8^+^ T cell effector functions, with CD103^+^ DCs requisite for complete effector differentiation, defined by expression of standard effector markers and their potential to enter inflamed tissues (CD25^hi^, T-bet^hi^, and Blimp-1^hi^ and CD62L^lo^ CCR5^hi^). In contrast, CD11b^hi^ DCs are more likely to prime CD8^+^ T cells, which largely remained in the lymph nodes, expressing molecules associated with the development of T_CM_ (CD62L^hi^, T-bet^lo^, Blimp-1^lo^ CD25^lo^, and CD127^hi^ (25). Thus, as entry into peripheral tissues is a defining characteristic of T_RM_ cells, it is likely that T_RM_ precursors are activated in the draining MdLN by activated respiratory CD103^+^ DCs, where they not only acquire effector function, but more importantly, the ability to accumulate in lung tissue, which is requisite for T_RM_ development. Priming by CD103^+^ DCs may also be one of the reasons that T_RM_ have a propensity to develop following induction of the responses in mucosal tissues, as similar CD103^+^ epidermal associated DCs are found predominately in these sites (26) and may be a common method promoting CD8^+^ T cell migration into peripheral tissues. In support of this, intranasal vaccination gives rise to populations of long lasting T_mem_ in the female reproductive tract, a phenomenon that is not observed following systemic infection (27–29). This indicates that a common priming requirement (possibly CD103^+^ DCs) can induce CD8^+^ T cell migration into more restrictive sites, and vaccination at certain mucosal surfaces may broadly confer protection at expanded peripheral sites (30). However, it should be noted that certain systemic infections, such as lymphocytic choriomeningitis virus (LCMV), can produce populations of T_RM_ cells in a wide variety of tissues, including the intestinal tract, brain, and female reproductive tract, as well as organs such as the kidney, heart, and pancreas, although in this study respiratory T_RM_ were not assessed (31). LCMV, a true systemic pathogen, can replicate in multiple cell types and locations, suggesting that pathogen promiscuity could result in activation of CD103^+^ DC and induction of T_RM_ independent of mucosal infection. For the lung, it seems that priming via the respiratory route [intranasally (i.n.)] is necessary for T_RM_ formation, as priming with influenza virus intraperitoneally (i.p.) fails to generate T_RM_ cells (20). The difference here is that influenza will not produce a productive infection when given by the i.p. route, limiting presentation to CD103^−^ DCs (32). It will be important for future studies to distinguish whether the lung is truly a restrictive site, limiting T_RM_ generation only after infection via the i.n. route, or if a systemic or mucosal challenge at a divergent site can induce lung T_RM_ populations under the right conditions.

Another important factor which can be highly variable during infection is the presence of particular cytokines, which influence both memory cell potential (33, 34), and the specific pool of T_mem_ that develops (35). The potential for an effector T cell (T_eff_) to become a T_mem_ cell has been defined based on the expression of CD127 and KLRG1 (36). T_effs_ largely fall into one of three categories: terminally differentiated short-lived effector cells (SLECs, KLRG1^hi^/CD127^lo^), early effector cells (EECs, KLRG1^lo^/CD127^lo^) or memory precursor effector cells (MPECs, KLRG1^lo^, CD127^hi^). It is the latter population, which develops into long-lived, bonafide T_mem_ of various phenotypes, including T_CM_, T_EM_, and T_RM_. Therefore, the formation of MPECs is a necessary step in T_RM_ development, although the timing in which a cell begins to express these markers may differentially impact its memory phenotype. MPECs can form early in the lymph node, or once at the site of infection they can arise from EECs, which have the potential to differentiate into both SLECs and MPECs (37). The inflammatory cytokine IL-12 is detectable at 48 h following influenza infection, and is important for the development of IFN-γ producing cells early in the immune response (38). In regard to memory development, IL-12 promotes the development of terminally differentiated SLECs in a dose dependent manner via induction of the transcription factor T-bet (33). Interestingly, graded induction of IL-12 is observed after systemic infection with two different pathogens: *L. monocytogene*s (LM) induces a high concentration of IL-12, whereas vesicular stomatitis virus (VSV) induces much lower IL-12 levels. High concentrations of IL-12 during LM infection promote a skewed development favoring SLECs while VSV infection (lower IL-12) favored EECs (37). Since T_RM_ cells arise from KLRG1^lo^ precursors (14), high levels of IL-12 would likely negatively impact T_RM_ development. Nonetheless, a minimum threshold of IL-12 (and T-bet) expression is required to not only promote the requisite development of T_eff_ but promote migration into peripheral sites. In support of this, it has been shown that CD103^+^ DCs isolated from the small intestine are capable of producing IL-12 following TLR stimulation (39). However, high levels of IL-12 signaling had a direct effect on CD8^+^ T cells, leading to the down-regulation of CXCR3, a molecule necessary for the accumulation of antigen-specific CD8^+^ T cells in the airways following influenza infection (40). These data would suggest that CD8^+^ T cells at the site of priming need just the right amount of IL-12 to reach their full T_RM_ potential. In terms of cytokines important for parsing T_mem_ into defined subsets, the common gamma chain cytokines IL-2 and IL-15 have been shown to play a role in CD8^+^ T cell differentiation into T_CM_ and T_EM_ cells. T_CM_ cells can be identified as a distinct population arising from MPECs as early as 5 dpi, and are formed through IL-15 signaling (when IL-2 is limited), whereas IL-2 signaling leads to T_EM_ phenotypes (41). As previously mentioned T_CM_ cells develop early after infection from the MPEC population in the lymph node, and these cells may never enter peripheral tissues. Thus, T_RM_ cells may arise from T_effs_, which do not receive early T_CM_ biasing signals in the lymph node, and retain the ability to enter peripheral sites.

While the evidence we have presented thus far suggests that specific cellular interactions and cytokines present in the lymph node at the time of priming could form a population of cells with the potential to become T_RM_ cells, an early development pathway completely unique to T_RM_ remains unlikely. Traditional cell surface markers and functional characteristics associated with T_eff_ cells or T_EM_, such as low levels of CD62L expression, are indistinguishable from T_RM_ early after infection. Moreover, the prototypical T_RM_ cell surface marker, CD103, does not appear until after a certain period of tissue residency in the epidermis (14). Interestingly, T_RM_ populations in the skin require the expression of CXCR3 for entry into the epithelium and subsequent T_RM_ differentiation as cells lacking CXCR3 remained largely outside of the epidermis and T_RM_ recovered from the skin were numerically reduced. Conversely, mice lacking CCR7 expression have CD8^+^ T_effs_, which fail to leave the skin via the lymphatics and harbor larger numbers of T_RM_ cells, suggesting environmental factors are required for complete T_RM_ development (14). Signals encountered in the MdLN after respiratory infection likely generate a population of T_eff_ cells with the potential to enter the lung and fully develop into true T_RM_ cells. However, CD8^+^ T cells isolated from the respiratory tract phenotypically resemble T_EM_ cells, T_RM_ cells, and terminally differentiated SLECs (our unpublished observations), suggesting not all T_eff_ that enter the lung become T_RM_. More likely, cells immigrating to the respiratory tract enter as a common T_EM/RM_ precursor (Figure 1A). This common T_EM/RM_ precursor population is likely primed by CD103^+^ DCs, expresses high levels of CD25, and encounters intermediate levels of IL-12, akin to the development of T_EM_ cells. Therefore, T_RM_ and T_EM_ cells may share similar early developmental pathways, with later signals in the lung further differentiating and diverting true T_RM_ from a common T_EM/RM_ precursor. Indeed, evidence supports the development of a common T_EM_/T_RM_ precursor. As previously noted, the development of T_EM_ cells is dependent on IL-2 (and not IL-15) (41) and IL-15 is also dispensable for CD8^+^ T_mem_ that develops following a respiratory infection (42), which generates substantial T_RM_ compared to systemic infection (20). In contrast, systemic infections produce large amounts of T_CM_ cells, and T_mem_ in these infections require IL-15 for maintenance over time (43). Although the evidence suggests a common developmental pathway for T_EM_ and T_RM_ cells after initial activation in the lymph node, the possibility remains that they are distinct lineages by the time of lymph node egress (Figure 1B), identifiable by phenotypic markers or gene expression patterns yet to be discovered. Nonetheless, the full commitment to the T_RM_ lineage will continue in the specific peripheral tissue, where these cells will be retained.

### Environmental signals commit T_EM_/T_RM_ precursor cells to a T_RM_ lineage

If T cell priming in the MdLN results in the migration of a common T_EM/RM_ precursor population of cells to the lung, what factors in the lung facilitate the development of “full-fledged” T_RM_ cells? At the site of infection multiple factors will continue to influence emigrating T_EM/RM_ precursors. Evidence in cutaneous infection models suggests that commitment to the T_RM_ lineage is a two-step process characterized by the sequential up-regulation of Bcl-2 and CD69, followed by CD103 (14). This suggests that T cells first acquire a memory phenotype, or an increased chance of survival, prior to differentiating into T_RM_ cells based on the current T_RM_ phenotypic markers. This section will discuss the respiratory factors that influence the transition to a memory phenotype and specific environmental components present in the lung that polarize these anti-viral CD8^+^ T cells toward a T_RM_ lineage.

#### The inflammatory environment of the lung

The pioneer T_eff_ cells immigrating to the lung arrive ~5–6 days after initial respiratory infection. Prior to their arrival, innate immune cells have accumulated, keeping viral titers low, and as a result, some local tissue damage has occurred via cytolysis of infected epithelial cells, affecting barrier function. The inflammatory effects of this local immune response in the lung are still very present at the time of T cell entry, and can influence the development of T_RM_ cells. However, since anti-influenza T_eff_ migrate to the lung asynchronously over several days (peaking at ~10 days post viral infection), all T cells do not encounter equivalent levels of inflammation which will likely affect the fate of individual T_eff_ clones.

The first CD8^+^ T_effs_ to arrive at the site of infection will encounter the greatest level of inflammation, as infectious virus is still present (at least until ~d8 post influenza infection) and innate effectors such as NK cells are producing local IFN-γ (38). Inflammatory monocyte-derived DCs arrive in the inflamed lung at the same time as T_eff_ and function as lung APCs, amplifying the inflammatory milieu and locally expanding the emigrating T_eff_ (44). Additionally, CD8^+^ T cell proliferation continues in the lung, a process requisite for viral control after influenza infection (45). This additional expansion, however, is not without a cost. Increased levels of cellular division is not only associated with increased levels of apoptosis within the highly dividing populations (46), the aforementioned cytokines also promote terminal differentiation of the T cells and the formation of KLRG1^+^ SLECs (47, 48). Therefore, this early inflammatory environment skews cells away from becoming memory cells, yet may paradoxically pave the way for resolution from infection and inflammation so that later immigrants may develop into T_mem_.

CD8^+^ T_eff_ themselves produce cytokines in the lung, including IL-2, IFN-γ and TNF-α which enhance the overall inflammatory response (49). Interestingly, while CD8^+^ T cells activated in lymph nodes rapidly gain the ability to produce the inflammatory cytokine IFN-γ, entry into the lung tissue imparts IL-10 production (50, 51) in a manner seemingly dependent on the inflammatory lung environment (52), indicating that an enhanced activation status resulting from high levels of inflammation induces the CD8^+^ T cells to produce regulatory cytokines. IL-10 is also produced at high levels by regulatory T cells (T_regs_) activated in the lung following influenza infection (53). The production of regulatory cytokines by T_regs_ and CD8^+^ T cells is important to initiate “dampening” the immune responses in the lung to prevent excessive damage and loss of function of this essential organ. Importantly, the production of IL-10 can directly impact the development of memory cells by inducing MPEC populations in a STAT3 dependent manner (54), however, it is unclear whether IL-10 has any direct consequences on the development of T_RM._

A variety of other cytokines produced after influenza infection is known to modulate anti-viral CD8^+^ T cell responses. Thymic stromal lymphopoietin (TSLP), an epithelial derived cytokine that can be produced in the infected lung (55, 56), promotes expansion of the CD8^+^ T cells at the site of infection directly (56) and indirectly via CD11b^+^ inflammatory DCs (57). Additionally, transpresentation of IL-15 by pulmonary DCs has been shown to increase the survival of T_effs_ (58) and is an important component of T_RM_ development in the skin (14). However, IL-15 does not seem necessary for the overall development of memory in the lungs or the airways following influenza infection (42), although this study as well as the TSLP studies did not address T_RM_ populations specifically.

#### Localization as an important step in development of T_RM_ cells

As residence at the peripheral site is a requisite for T_RM_ development, cells destined to become T_RM_ cells must first gain access into peripheral tissues, often into physically restricted areas such as within an epithelial layer or closely associated with the underlying basil lamina (within the parenchyma). The route of migration used by T cells trafficking to the lung, however, is not well understood. Cells can enter the lung via two circulatory systems: the bronchial system, which provides oxygenated blood to the lung tissue, and the pulmonary circulation, which includes vessels that bring deoxygenated blood to alveoli and subsequently drain oxygenated blood back to the heart (59). The lung epithelium surrounding the airway spaces share a fused basal lamina with the adjacent capillary endothelium to allow gas exchange and could facilitate direct blood to airway traffic. Because pulmonary vessels are small in diameter and thin walled, blood pressure in these vessels is relatively low, thus allowing lymphocytes to traverse the endothelium independent of the multistep paradigm described for lymphocyte migration through larger vessels, which are dependent on selectins, integrins, and chemokines (60). However, histological sections of lung tissues depict memory cells localized close to the airways, but within the lung parenchyma, evoking a blood → lung → airway route (19, 61).

Broadly speaking, activated CD8^+^ T_effs_ cells can gain access to peripheral sites by virtue of their expression of CD11a and CD44 with concomitant loss of CD62L expression on their cell surface (60, 62).While access into distinct anatomical sites within other mucosal tissues such as the skin and gut is highly correlated with expression of tissue-specific homing receptors (63–65), analogous molecules have not yet been identified for lung homing CD8^+^ T cells. Nonetheless, some chemotactic signals are associated with T_eff_ migration into inflamed lung tissues including CXCR3 (66) and CXCR6 (67). CXCR6 is specifically up-regulated on CD8^+^ T cells isolated from the lung and lung airways following intranasal immunization and mice lacking CXCR6 have reduced protection against tuberculosis challenge (67), indicating that CXCR6 expression may be important for the establishment of CD8^+^ T cells at sites of protection. The expression of CXCR3 is important to establish migration of CD8^+^ T cells specifically to the airways (68). While T_RM_ populations were not assessed in this study, CXCR3^−^ antigen-specific CD8^+^ T cells isolated from the lung expressed lower levels of CD69 than WT cells occupying the airways where antigen is present. CD69 expression is upregulated on T_RM_ populations, and contact with antigen has been suggested to be necessary for T_RM_ formation (8). Therefore, expression of CXCR3 may be a requirement for the development of T_RM_ cells in the lungs, akin to the requirement for CXCR3 in the skin (14).

As influenza virus replicates primarily in epithelial tissue, the localization of CD8^+^ T cells adjacent to antigen may expose them to unique cytokines available in and near the epithelium such as TGF-β. TGF-β plays a role in both the contraction of effector T cells (69) and the establishment of T_RM_ cells by inducing the expression of CD103 (70). The role of TGF-β in the development of T_RM_ cells has been well described in the intestinal mucosa and the skin, and has also been implicated in the development of T_RM_ in the lung (71). Although TGF-β can be transiently activated by influenza virus (72, 73), it likely has lower constitutive production in the lung than other barrier sites as over-expression of TGF-β can promote pulmonary fibrosis and lung disease (74). Due to the localization of TGF-β production, CD103 expression may be specific to only those cells, which are found within epithelial layers and not necessary for T_RM_ in the lung parenchyma, a concept discussed later in more detail. Interestingly, following influenza infection a large majority of antigen-specific CD8^+^ T cells begin to express the α1β1 integrin VLA-1 (61). T_effs_ localized cells to the collagen-rich areas near the airways and basement membranes that are VLA-1^+^ have a survival advantage over those that do not express VLA-1 at the peak of the CD8^+^ T cell response (61). The localization and retention of cells within the lung parenchyma, as well as the survival advantage may make VLA-1 expression a unique marker for cells destined to become lung T_RM_ cells. However, this possibility has yet to be explored.

## Part II: Characteristics and Maintenance of Committed CD8^+^ T_RM_ in the Lung

Following the resolution from infection, antigen-specific CD8^+^ T cells will persist at the site of infection (19). As previously noted, these T_mem_ cells exist in the lung in two basic compartments, the airways and the lung parenchyma. Airway CD8^+^ T cells exist outside of the body, within the lumen of the respiratory tract, or they can exist much like they do in the intestinal epithelium as intraepithelial cells. Cells within the airways, and very likely some intraepithelial cells, can be isolated by performing a bronchoalveolar lavage (BAL), while the remaining parenchyma cells can be isolated through a process involving the enzymatic digestion of collagen. Additionally the localization and characterization of these cell populations can be defined by microscopic analysis of lung tissue sections, although phenotyping cells by this method is limited. It is important to distinguish between these two populations of cells in the discussion of T_RM_, as airway cells are likely comprised of both true T_RM_ cells and circulating T_mem_, which migrate to the airways following the resolution of infection.

Cells in the airways are subject to the external environment of the lung, where mucous and pulmonary surfactants decrease the potential for their long-term persistence. Therefore, it is thought that memory CD8^+^ T cells in the lung airways, at least for some period of time, are partially maintained by the continual recruitment to the airways. In support of this, Slutter et al. showed that CXCR3 is required for the continual recruitment of cells into the airways, and that loss of CXCR3 expression results in the accelerated loss of antigen-specific CD8^+^ T cells specifically from the airways (40). Tracking the entry of T_mem_ from the circulation is also possible by monitoring CD11a expression, which is lost ~40 h after CD8^+^ T cell emigration into the airways (75). Indeed, when T_mem_ are extracted from the airways (up until at least 13 months post infection), portions of the antigen-specific CD8^+^ T cells express high levels of CD11a. Together, these data confirm that at least a proportion of airway CD8^+^ cells may not be bona fide T_RM_ based on presence within this site alone. In support of this argument, CD103 expression is reduced on antigen-specific T_mem_ isolated from the airway when compared to T_mem_ isolated from the lung parenchyma both in terms of frequency (11) and on a per cell basis (76). Finally, while evidence suggests that a circulating population of cells is actively recruited into the lung airways during steady state conditions (40, 75) it is clear that these recruits are not sufficient (either in number or function) to provide protection against heterosubtypic influenza challenge, as protection wanes while recruitment continues. Perhaps the limited migration and supplementation of competent T_mem_ cells from within the lung parenchyma may augment this pool and maintain heterosubtypic immunity, at least temporarily. However, cell tracking studies have not confirmed this possibility.

T_mem_ also exist in the respiratory tract within the lung tissue or parenchyma. As the lung is a highly vascularized organ, it can be difficult to discern at time of tissue harvest, which antigen-specific cells are trafficking through the vasculature of the lung (trapped within small capillaries) and which are truly within the parenchyma. Experiments using intravascular staining whereby antibodies are injected directly into the blood stream immediately before the lungs are examined to “tag” circulating cells demonstrated a large number of cells isolated from the lung tissue are circulating cells (naïve or T_EM_) despite perfusion. This method has been useful in characterizing both CD4^+^ (77, 78) and CD8^+^ (11) T_RM_ cells in direct contrast to the circulating pool. Using this method to distinguish circulating vs. resident cells has, and will, continue to provide a clearer picture of what T_RM_ cells look like in the resting lung.

Microscopic analysis of lung tissue sections has also been useful in determining the precise localization of T_RM_ in the respiratory tract to gain better insight regarding the cellular associations and tissue microarchitecture, which may be important for supporting T_RM_ development and/or survival. Turner et al. showed that CD4^+^ T_RM_ cells established following influenza infection were clustered together in the lungs, in regions both close to the airways and to the pulmonary blood vessels (78). This would position the cells in an ideal place to encounter antigen entering the body. The clustering of cells in this location is not a new observation, nor is it exclusive for the CD4^+^ T cell population. In 2004, Ray et al. showed that influenza specific CD8^+^ T cells persisted in the highly collagenized area between the airways and the blood vessels, and that this retention was dependent on the expression of VLA-1 (61). VLA-1 binds to type IV and type I collagen (79, 80), which are important structural components of the lung interstitium, specifically between the bronchi and the vasculature, and the basement membranes of both the pulmonary vasculature and the epithelium of the airway, respectively (81, 82). The co-localization of T_RM_ and collagen below the epidermal cell layer of the airways shows that T_RM_ cells also exist within the lung parenchyma. The collagen-rich environment of the lung may provide a framework or scaffold in which T_RM_ cells can persist close to the site of antigen acquisition, yet not actually within the epithelial layer of the lung where they may be subject to the harsh environment of the airways. Additionally, it is quite possible that this collagen matrix could also trap or capture soluble growth factors important for T_RM_ maintenance.

### The persistence of T_RM_ cells in the respiratory tract: Role of the lung environment

Like other mucosal barrier sites, the resting lung is engaged in a constant balancing act regarding immunity and tolerance. It is estimated that we breathe in 10,000 l of air per day, with each breath containing a plethora of allergens, environmental pollutants, and pathogens. Inappropriate response to non-harmful antigens could lead to persistent inflammation and pulmonary disease. To prevent this, multiple layers of innate protection exist in the lung to preclude any inappropriate initiation of an immune response. The most basic of these is the mucosal barrier itself. The lining of the upper respiratory tract is composed of ciliated epithelial cells and mucus-secreting goblet cells, which together function as a “mucociliary escalator” facilitating expulsion of these innocuous agents, as well as some commensal organisms, out of the respiratory tract without activation of the adaptive immune response. However, the mucus would also prevent T_RM_ cells from persisting in the airways of the upper respiratory tract, leading to the accumulation of T_RM_ either within the epithelium, the parenchyma, or in the airways of the lower respiratory tract. While the lower respiratory tract does not contain mucous, it is characterized by numerous “pockets” where gas exchange occurs termed alveoli. The cells lining the alveoli are specialized epithelial cells known as type I and type II alveolar epithelial cells, which form the structural architecture of the alveoli and secrete immunosuppressive pulmonary surfactants, respectively (83). The role that these lung derived factors may play on CD8^+^ T cells at the site is further complicated by conditions of an inflamed lung, such as asthma and allergy. Allergens can induce the upregulation of pulmonary surfactants, which in turn can protect against allergic disease via local IL-13 inhibition (84). Due to the proximity of surfactants and T_RM_ cells in the lower respiratory tract, and the essential role for surfactants in regulating respiratory inflammation, it is possible that T_RM_ persistence could be dynamically regulated by perturbation in surfactant (and mucus) activity. However, this has not been analyzed.

T_RM_ persist long-term in many non-lymphoid tissues, albeit with different kinetics. For example, VSV-specific T_RM_ cells exist as long as 120 dpi in the brain (8) while cutaneous herpes simplex virus T_RM_ cells persist for the lifetime of a mouse (85). This is shown to occur independently of increased proliferation (8, 15) and maintained populations are not dependent on replenishment from lymphoid organs (6, 78). Perhaps somewhat unique to the respiratory tract is that T_mem_ cells within this site appear to have a limited life-span, steadily decreasing over time (19). The lack of long-term survival of T_mem_ cells lung airways, and perhaps certain populations in the lung itself, has functional consequences since heterosubtypic immunity against influenza viruses is lost ~4–6 months post infection (18). Moreover, this loss of anti-influenza immunity is coordinate with substantial loss in CD8^+^ T_mem_ cells of the airways, despite stable numbers in the spleen (19) and the continual recruitment of cells from the circulation into the airways (40, 75). While these former studies did not directly assess the role of T_RM_ cells, recent evidence suggests that protective heterosubtypic immunity against influenza infection is mediated solely by T_RM_, as the ability to control viral titers and protect from severe disease is gradually lost along with T_RM_ cells in the airways (20). Yet, the question of why T_RM_ cells do not persist in the lung and lung airways to the extent that they do in other tissues remains unanswered. Interestingly, following influenza infection lung T_RM_ cells retain expression of interferon-induced transmembrane protein IFITM3, which imparts cells with a survival advantage in the face of viral infection (76). This increased survival mechanism may be particularly important at this site, due to the regularity at which respiratory infections are acquired. The unique properties of respiratory T_RM_ cells have provided some insight into why their persistence in the lungs is limited (Table 1).

**Table 1:** **Factors associated with the positioning and survival of defined pools of memory CD8^+^ T cells in specific anatomical sites**.

	CD127	CD122	PD-1	CD103	CXCR3	IFITM3	CD69	CD27	VLA-1
T_EM_	**+++**	**+++**	**−**	**−**		**−/+**	**−**	**+**	
T_CM_	**+++**	**+++**	**−**	**−**		**−/+**	**−**	**++**	
T_RM_ Lung	**−/+**	**+**	**++**	**−/+**	**+++**	**+++**	**+++**	**++/+++**	**+++**
T_RM_ Gut	**+/++**	**+**		**+++**			**+++**	**+**	++*
T_RM_ Skin	**+**	**+**		**+++**	**+++**		**+++**		+*
T_RM_ Brain	**++**	**+**	**++**	**+++**		**+++**	**+++**		**−**

***−**absent, +low levels, ++moderate levels, +++high levels, blank = no data for this tissue*.

*Indicates data is from human studies, all other data in table obtained from mouse models.

The cytokines IL-7 and IL-15 are requisite for the development and maintenance of memory CD8^+^ T cells after systemic infection (35, 86). However, what role, if any, these cytokines play in the maintenance of T_RM_ cells in the lung has not been defined. In most sites assessed to date, T_RM_ cells express reduced levels of CD127, as compared to T_CM_ and T_EM_ cells. Concurrently, CD8^+^ T cells in the lung airways express reduced levels of CD127 (11, 87, 88) as do cells in the lung parenchyma, although to a lesser extent (56). Like CD11a, it is possible that CD127 is cleaved from CD8^+^ T cells in the airways, leaving these cells incapable of receiving proliferative or survival signals, either from IL-7 or from TSLP, which has been shown to be produced constitutively in the gut (89), and in the lung during both resting conditions and after inflammatory stimuli (56). IL-15 has been shown to be dispensable for the development and maintenance of memory cells that develop from respiratory infections and CD122 is lost from CD8^+^ T cells within the respiratory tract (87). Furthermore, CD122 or the beta chain of the IL-15R, which signals to memory CD8^+^ T cells is expressed at lower levels on T_RM_ isolated from the epithelium of the small intestine (90). A recently described pool of T_RM_ isolated from secondary lymphoid organs are maintained independently of IL-15 and even found in increased numbers in mice lacking IL-15 (91). Therefore, IL-15 appears to be uniformly dispensable for the maintenance of T_RM_ cells, and while levels of CD127 on T_RM_ cells is more variable, the near complete loss of this receptor in the respiratory tract may provide one mechanism in which CD8^+^ T cells at this site have decreased sustainability. However, it should be noted that T_RM_ cells from the brain do not respond to IL-7 or IL-15 *ex vivo*, unlike splenic memory cells, which show increased survival upon exposure to these cytokines (16), indicating that perhaps the survival of T_RM_ cells is completely independent of classical cytokine memory signals.

The maintenance of CD8^+^ T cells in the lungs has also been attributed to residual antigen found in the MdLN for ~2 months post influenza infection (92). Influenza antigens have also been detected in the lung tissue itself for 30 days within focal inflammatory structures (93), reminiscent of inducible bronchus associated lymphoid tissue (iBALT). iBALT develops following influenza infection and has similar structure to lymph node tissue, such as defined B cell follicles and the formation of germinal centers surrounding DCs; this structure contributes to the proliferation of B and T cells during primary influenza infection and can be protective in mice where other lymphoid organs are lacking (94). As the timing of loss of residual antigen coincides with the loss of protective heterosubtypic immunity, it has been hypothesized that antigen is necessary for the persistence of T_mem_ in the lung and lung airways. In support of this possibility, T_RM_ cells in the lung express PD-1 (20), which may indicate continued exposure to antigen. While certain T_RM_ populations have been shown to persist in the absence of antigen (8, 31) definitive studies have not been carried out for T_RM_ cells in the lung to rule this out as a mechanism for maintenance.

It is likely in humans that the maintenance and survival of T_RM_ cells may be much different than what is observed in mice. As previously mentioned, constant antigenic stimulation, allergic inflammation, and relatively common airway disorders such as asthma will influence the lung environment in ways that will affect many indigenous respiratory cells. In addition, the regularity of respiratory infections in humans will result in the accumulation of many pools of clonally diverse antigen-specific cells, recognizing a plethora of pathogens. de Bree et al. showed that influenza and respiratory syncytial virus-specific CD8^+^ T cells were enriched in the human lung compared to the circulation (95). In direct contrast, antigen-specific CD8^+^ T cells that developed from the blood-borne pathogens cytomegalovirus and Epstein-Barr virus equilibrated between the blood and lung of these patients (95). The accumulation of CD8^+^ T cells in the lung due to respiratory infection would certainly lead to large numbers of T_RM_ cells populating the human lung during steady state conditions. Indeed, studies have determined that CD103^+^αβ TCR CD8^+^ T cells comprise about 1/3 of the total CD8^+^ T cell population in the human lung (96), or over 10 billion total cells (97). However, the history of human lung T_RM_ (when developed/how long maintained) and how the history of individual clones correlates with acquisition of specific infections is difficult to determine. Furthermore, in humans, the survival of these pools may be affected by attrition resulting from heterologous infections. In these scenarios, either competition for resources in distinct environmental niches or by bystander apoptosis via cytotoxic factors present at the time of the new viral infection may deplete previously existent T_RM_ pools (98).

## Concluding Remarks

The study of T_RM_ cells is in its infancy. As we continue to analyze this unique lineage of memory cells, we will certainly deepen our understanding of T_RM_ biology in unique sites such as the respiratory tract and perhaps better understand how to selectively manipulate this pool for development of vaccines. While the defining characteristic of what makes a cell a T_RM_ cell is quite clear (i.e., long-term residence at a site), some of the markers currently used to distinguish T_RM_ cells, most notably CD103, only recognize a subset of T_RM_ cells localized to the (respiratory) epithelium. This leaves a large population (anywhere from 50 to 90% of T_RM_ cells in the lung) excluded from studies. Thus, overall T_RM_ frequency can only be confirmed using complicated transfer and cell tracking experiments, warranting the need for more definitive phenotypic markers to readily identify T_RM_. Moreover, understanding the environment in which T_RM_ cells at specific sites reside will be key to developing phenotypic definitions of these cells, as markers vary between anatomical locations. In the case of the lung, this particular environment has many mechanisms in place to suppress inflammation and any inadvertent immunopathology. Thus, while higher numbers of T_RM_ cells at the site of infection may be ideal for protection against disease, tight regulation of the number, and longevity of T_RM_ cells at this site may be essential for tissue function. This may be especially relevant in the context of human disease, where respiratory infections are commonplace and populations of T_RM_ are not only numerically enhanced but very likely dynamically regulated.

## Conflict of Interest Statement

The authors declare that the research was conducted in the absence of any commercial or financial relationships that could be construed as a potential conflict of interest.

## References

[1] Woodland DL Hogan RJ Zhong W Cellular immunity and memory to respiratory virus infections. Immunol Res (2001) 24(1):53–67 10.1385/IR:24:1:53 11485209

[2] Sallusto F Lenig D Forster R Lipp M Lanzavecchia A Two subsets of memory T lymphocytes with distinct homing potentials and effector functions. Nature (1999) 401(6754):708–12 10.1038/44385 10537110

[3] Pham TH Okada T Matloubian M Lo CG Cyster JG S1P1 receptor signaling overrides retention mediated by G alpha i-coupled receptors to promote T cell egress. Immunity (2008) 28(1):122–33 10.1016/j.immuni.2007.11.017 18164221 PMC2691390

[4] Klonowski KD Williams KJ Marzo AL Blair DA Lingenheld EG Lefrancois L Dynamics of blood-borne CD8 memory T cell migration in vivo. Immunity (2004) 20(5):551–62 10.1016/S1074-7613(04)00103-7 15142524

[5] Masopust D Vezys V Usherwood EJ Cauley LS Olson S Marzo AL Activated primary and memory CD8 T cells migrate to nonlymphoid tissues regardless of site of activation or tissue of origin. J Immunol (2004) 172(8):4875–82 10.4049/jimmunol.172.8.4875 15067066

[6] Masopust D Choo D Vezys V Wherry EJ Duraiswamy J Akondy R Dynamic T cell migration program provides resident memory within intestinal epithelium. J Exp Med (2010) 207(3):553–64 10.1084/jem.20090858 20156972 PMC2839151

[7] Jiang X Clark RA Liu L Wagers AJ Fuhlbrigge RC Kupper TS Skin infection generates non-migratory memory CD8+ T(RM) cells providing global skin immunity. Nature (2012) 483(7388):227–31 10.1038/nature10851 22388819 PMC3437663

[8] Wakim LM Woodward-Davis A Bevan MJ Memory T cells persisting within the brain after local infection show functional adaptations to their tissue of residence. Proc Natl Acad Sci U S A (2010) 107(42):17872–9 10.1073/pnas.1010201107 20923878 PMC2964240

[9] Shin H Iwasaki A A vaccine strategy that protects against genital herpes by establishing local memory T cells. Nature (2012) 491(7424):463–7 10.1038/nature11522 23075848 PMC3499630

[10] Mackay LK Stock AT Ma JZ Jones CM Kent SJ Mueller SN Long-lived epithelial immunity by tissue-resident memory T (TRM) cells in the absence of persisting local antigen presentation. Proc Natl Acad Sci U S A (2012) 109(18):7037–42 10.1073/pnas.1202288109 22509047 PMC3344960

[11] Anderson KG Sung H Skon CN Lefrancois L Deisinger A Vezys V Cutting edge: intravascular staining redefines lung CD8 T cell responses. J Immunol (2012) 189(6):2702–6 10.4049/jimmunol.1201682 22896631 PMC3436991

[12] Ziegler SF Ramsdell F Alderson MR The activation antigen CD69. Stem Cells (1994) 12(5):456–65 10.1002/stem.5530120502 7804122

[13] Schon MP Arya A Murphy EA Adams CM Strauch UG Agace WW Mucosal T lymphocyte numbers are selectively reduced in integrin alpha E (CD103)-deficient mice. J Immunol (1999) 162(11):6641–9 10352281

[14] Mackay LK Rahimpour A Ma JZ Collins N Stock AT Hafon ML The developmental pathway for CD103(+)CD8+ tissue-resident memory T cells of skin. Nat Immunol (2013) 14(12):1294–301 10.1038/ni.2744 24162776

[15] Gebhardt T Wakim LM Eidsmo L Reading PC Heath WR Carbone FR Memory T cells in nonlymphoid tissue that provide enhanced local immunity during infection with herpes simplex virus. Nat Immunol (2009) 10(5):524–30 10.1038/ni.1718 19305395

[16] Wakim LM Woodward-Davis A Liu R Hu Y Villadangos J Smyth G The molecular signature of tissue resident memory CD8 T cells isolated from the brain. J Immunol (2012) 189(7):3462–71 10.4049/jimmunol.1201305 22922816 PMC3884813

[17] Sheridan BS Pham QM Lee YT Cauley LS Puddington L Lefrancois L Oral infection drives a distinct population of intestinal resident memory CD8(+) T cells with enhanced protective function. Immunity (2014) 40(5):747–57 10.1016/j.immuni.2014.03.007 24792910 PMC4045016

[18] Liang S Mozdzanowska K Palladino G Gerhard W Heterosubtypic immunity to influenza type A virus in mice. Effector mechanisms and their longevity. J Immunol (1994) 152(4):1653–61 8120375

[19] Hogan RJ Usherwood EJ Zhong W Roberts AA Dutton RW Harmsen AG Activated antigen-specific CD8+ T cells persist in the lungs following recovery from respiratory virus infections. J Immunol (2001) 166(3):1813–22 10.4049/jimmunol.166.3.1813 11160228

[20] Wu T Hu Y Lee YT Bouchard KR Benechet A Khanna K Lung-resident memory CD8 T cells (TRM) are indispensable for optimal cross-protection against pulmonary virus infection. J Leukoc Biol (2014) 95(2):215–24 10.1189/jlb.0313180 24006506 PMC3896663

[21] Obar JJ Lefrancois L Early events governing memory CD8+ T-cell differentiation. Int Immunol (2010) 22(8):619–25 10.1093/intimm/dxq053 20504887 PMC2908475

[22] Mescher MF Curtsinger JM Agarwal P Casey KA Gerner M Hammerbeck CD Signals required for programming effector and memory development by CD8+ T cells. Immunol Rev (2006) 211:81–92 10.1111/j.0105-2896.2006.00382.x 16824119

[23] Youngblood B Hale JS Ahmed R T-cell memory differentiation: insights from transcriptional signatures and epigenetics. Immunology (2013) 139(3):277–84 10.1111/imm.12074 23347146 PMC3701173

[24] Kim TS Braciale TJ Respiratory dendritic cell subsets differ in their capacity to support the induction of virus-specific cytotoxic CD8+ T cell responses. PLoS One (2009) 4(1):e4204 10.1371/journal.pone.0004204 19145246 PMC2615220

[25] Kim TS Gorski SA Hahn S Murphy KM Braciale TJ Distinct dendritic cell subsets dictate the fate decision between effector and memory CD8(+) T cell differentiation by a CD24-dependent mechanism. Immunity (2014) 40(3):400–13 10.1016/j.immuni.2014.02.004 24631155 PMC4017923

[26] del Rio ML Bernhardt G Rodriguez-Barbosa JI Forster R Development and functional specialization of CD103+ dendritic cells. Immunol Rev (2010) 234(1):268–81 10.1111/j.0105-2896.2009.00874.x 20193025

[27] Gallichan WS Rosenthal KL Long-lived cytotoxic T lymphocyte memory in mucosal tissues after mucosal but not systemic immunization. J Exp Med (1996) 184(5):1879–90 10.1084/jem.184.5.1879 8920875 PMC2192861

[28] Ferko B Stasakova J Sereinig S Romanova J Katinger D Niebler B Hyperattenuated recombinant influenza A virus nonstructural-protein-encoding vectors induce human immunodeficiency virus type 1 Nef-specific systemic and mucosal immune responses in mice. J Virol (2001) 75(19):8899–908 10.1128/JVI.75.19.8899-8908.2001 11533153 PMC114458

[29] Gherardi MM Perez-Jimenez E Najera JL Esteban M Induction of HIV immunity in the genital tract after intranasal delivery of a MVA vector: enhanced immunogenicity after DNA prime-modified vaccinia virus Ankara boost immunization schedule. J Immunol (2004) 172(10):6209–20 10.4049/jimmunol.172.10.6209 15128809

[30] Ruane D Brane L Reis BS Cheong C Poles J Do Y Lung dendritic cells induce migration of protective T cells to the gastrointestinal tract. J Exp Med (2013) 210(9):1871–88 10.1084/jem.20122762 23960190 PMC3754860

[31] Casey KA Fraser KA Schenkel JM Moran A Abt MC Beura LK Antigen-independent differentiation and maintenance of effector-like resident memory T cells in tissues. J Immunol (2012) 188(10):4866–75 10.4049/jimmunol.1200402 22504644 PMC3345065

[32] Venturi V Davenport MP Swan NG Doherty PC Kedzierska K Consequences of suboptimal priming are apparent for low-avidity T-cell responses. Immunol Cell Biol (2012) 90(2):216–23 10.1038/icb.2011.36 21556018

[33] Joshi NS Cui W Chandele A Lee HK Urso DR Hagman J Inflammation directs memory precursor and short-lived effector CD8(+) T cell fates via the graded expression of T-bet transcription factor. Immunity (2007) 27(2):281–95 10.1016/j.immuni.2007.07.010 17723218 PMC2034442

[34] Cui W Kaech SM Generation of effector CD8+ T cells and their conversion to memory T cells. Immunol Rev (2010) 236:151–66 10.1111/j.1600-065X.2010.00926.x 20636815 PMC4380273

[35] Schluns KS Lefrancois L Cytokine control of memory T-cell development and survival. Nat Rev Immunol (2003) 3(4):269–79 10.1038/nri1052 12669018

[36] Kaech SM Tan JT Wherry EJ Konieczny BT Surh CD Ahmed R Selective expression of the interleukin 7 receptor identifies effector CD8 T cells that give rise to long-lived memory cells. Nat Immunol (2003) 4(12):1191–8 10.1038/ni1009 14625547

[37] Obar JJ Jellison ER Sheridan BS Blair DA Pham QM Zickovich JM Pathogen-induced inflammatory environment controls effector and memory CD8+ T cell differentiation. J Immunol (2011) 187(10):4967–78 10.4049/jimmunol.1102335 21987662 PMC3208080

[38] Monteiro JM Harvey C Trinchieri G Role of interleukin-12 in primary influenza virus infection. J Virol (1998) 72(6):4825–31 9573248 10.1128/jvi.72.6.4825-4831.1998 PMC110027

[39] Fujimoto K Karuppuchamy T Takemura N Shimohigoshi M Machida T Haseda Y A new subset of CD103+CD8alpha+ dendritic cells in the small intestine expresses TLR3, TLR7, and TLR9 and induces Th1 response and CTL activity. J Immunol (2011) 186(11):6287–95 10.4049/jimmunol.1004036 21525388

[40] Slutter B Pewe LL Kaech SM Harty JT Lung airway-surveilling CXCR3(hi) memory CD8(+) T cells are critical for protection against influenza A virus. Immunity (2013) 39(5):939–48 10.1016/j.immuni.2013.09.013 24238342 PMC3872058

[41] Obar JJ Lefrancois L Early signals during CD8 T cell priming regulate the generation of central memory cells. J Immunol (2010) 185(1):263–72 10.4049/jimmunol.1000492 20519649 PMC2997352

[42] Verbist KC Field MB Klonowski KD Cutting edge: IL-15-independent maintenance of mucosally generated memory CD8 T cells. J Immunol (2011) 186(12):6667–71 10.4049/jimmunol.1004022 21572025 PMC3110618

[43] Schluns KS Williams K Ma A Zheng XX Lefrancois L Cutting edge: requirement for IL-15 in the generation of primary and memory antigen-specific CD8 T cells. J Immunol (2002) 168(10):4827–31 10.4049/jimmunol.168.10.4827 11994430

[44] Bedoui S Gebhardt T Interaction between dendritic cells and T cells during peripheral virus infections: a role for antigen presentation beyond lymphoid organs? Curr Opin Immunol (2011) 23(1):124–30 10.1016/j.coi.2010.11.001 21112755

[45] McGill J Legge KL Cutting edge: contribution of lung-resident T cell proliferation to the overall magnitude of the antigen-specific CD8 T cell response in the lungs following murine influenza virus infection. J Immunol (2009) 183(7):4177–81 10.4049/jimmunol.0901109 19767567 PMC2762786

[46] Fraser KA Schenkel JM Jameson SC Vezys V Masopust D Preexisting high frequencies of memory CD8+ T cells favor rapid memory differentiation and preservation of proliferative potential upon boosting. Immunity (2013) 39(1):171–83 10.1016/j.immuni.2013.07.003 23890070 PMC3979587

[47] Cui W Joshi NS Jiang A Kaech SM Effects of Signal 3 during CD8 T cell priming: bystander production of IL-12 enhances effector T cell expansion but promotes terminal differentiation. Vaccine (2009) 27(15):2177–87 10.1016/j.vaccine.2009.01.088 19201385 PMC2803112

[48] Kalia V Sarkar S Subramaniam S Haining WN Smith KA Ahmed R Prolonged interleukin-2Ralpha expression on virus-specific CD8+ T cells favors terminal-effector differentiation in vivo. Immunity (2010) 32(1):91–103 10.1016/j.immuni.2009.11.010 20096608

[49] Kohlmeier JE Woodland DL Immunity to respiratory viruses. Annu Rev Immunol (2009) 27:61–82 10.1146/annurev.immunol.021908.132625 18954284

[50] Sun J Madan R Karp CL Braciale TJ Effector T cells control lung inflammation during acute influenza virus infection by producing IL-10. Nat Med (2009) 15(3):277–84 10.1038/nm.1929 19234462 PMC2693210

[51] Palmer EM Holbrook BC Arimilli S Parks GD Alexander-Miller MA IFNgamma-producing, virus-specific CD8+ effector cells acquire the ability to produce IL-10 as a result of entry into the infected lung environment. Virology (2010) 404(2):225–30 10.1016/j.virol.2010.05.004 20627346 PMC2906694

[52] Sun J Dodd H Moser EK Sharma R Braciale TJ CD4+ T cell help and innate-derived IL-27 induce Blimp-1-dependent IL-10 production by antiviral CTLs. Nat Immunol (2011) 12(4):327–34 10.1038/ni.1996 21297642 PMC3079402

[53] Bedoya F Cheng GS Leibow A Zakhary N Weissler K Garcia V Viral antigen induces differentiation of Foxp3+ natural regulatory T cells in influenza virus-infected mice. J Immunol (2013) 190(12):6115–25 10.4049/jimmunol.1203302 23667113 PMC3703618

[54] Cui W Liu Y Weinstein JS Craft J Kaech SM An interleukin-21-interleukin-10-STAT3 pathway is critical for functional maturation of memory CD8+ T cells. Immunity (2011) 35(5):792–805 10.1016/j.immuni.2011.09.017 22118527 PMC3431922

[55] Plumb AW Patton DT Seo JH Loveday EK Jean F Ziegler SF Interleukin-7, but not thymic stromal lymphopoietin, plays a key role in the T cell response to influenza A virus. PLoS One (2012) 7(11):e50199 10.1371/journal.pone.0050199 23189186 PMC3506535

[56] Shane HL Klonowski KD A direct and nonredundant role for thymic stromal lymphopoietin on antiviral CD8 T cell responses in the respiratory mucosa. J Immunol (2014) 192(5):2261–70 10.4049/jimmunol.1302085 24489089 PMC3943877

[57] Yadava K Sichelstiel A Luescher IF Nicod LP Harris NL Marsland BJ TSLP promotes influenza-specific CD8+ T-cell responses by augmenting local inflammatory dendritic cell function. Mucosal Immunol (2013) 6(1):83–92 10.1038/mi.2012.50 22806096 PMC3534170

[58] McGill J Van Rooijen N Legge KL IL-15 trans-presentation by pulmonary dendritic cells promotes effector CD8 T cell survival during influenza virus infection. J Exp Med (2010) 207(3):521–34 10.1084/jem.20091711 20212069 PMC2839152

[59] Gray H Standring S Ellis H Berkovitz BKB Gray’s Anatomy: the Anatomical Basis of Clinical Practice. Edinburgh (2005).

[60] Miyasaka M Tanaka T Lymphocyte trafficking across high endothelial venules: dogmas and enigmas. Nat Rev Immunol (2004) 4(5):360–70 10.1038/nri1354 15122201

[61] Ray SJ Franki SN Pierce RH Dimitrova S Koteliansky V Sprague AG The collagen binding alpha1beta1 integrin VLA-1 regulates CD8 T cell-mediated immune protection against heterologous influenza infection. Immunity (2004) 20(2):167–79 10.1016/S1074-7613(04)00021-4 14975239

[62] Shin H Iwasaki A Tissue-resident memory T cells. Immunol Rev (2013) 255(1):165–81 10.1111/imr.12087 23947354 PMC3748618

[63] Masopust D Jiang J Shen H Lefrancois L Direct analysis of the dynamics of the intestinal mucosa CD8 T cell response to systemic virus infection. J Immunol (2001) 166(4):2348–56 10.4049/jimmunol.166.4.2348 11160292

[64] Lefrancois L Parker CM Olson S Muller W Wagner N Schon MP The role of beta7 integrins in CD8 T cell trafficking during an antiviral immune response. J Exp Med (1999) 189(10):1631–8 10.1084/jem.189.10.1631 10330442 PMC2193647

[65] Svensson M Marsal J Ericsson A Carramolino L Broden T Marquez G CCL25 mediates the localization of recently activated CD8alphabeta(+) lymphocytes to the small-intestinal mucosa. J Clin Invest (2002) 110(8):1113–21 10.1172/JCI0215988 12393847 PMC150799

[66] Fadel SA Bromley SK Medoff BD Luster AD CXCR3-deficiency protects influenza-infected CCR5-deficient mice from mortality. Eur J Immunol (2008) 38(12):3376–87 10.1002/eji.200838628 19039768 PMC2749081

[67] Lee LN Ronan EO de Lara C Franken KL Ottenhoff TH Tchilian EZ CXCR6 is a marker for protective antigen-specific cells in the lungs after intranasal immunization against Mycobacterium tuberculosis. Infect Immun (2011) 79(8):3328–37 10.1128/IAI.01133-10 21628524 PMC3147559

[68] Kohlmeier JE Reiley WW Perona-Wright G Freeman ML Yager EJ Connor LM Inflammatory chemokine receptors regulate CD8(+) T cell contraction and memory generation following infection. J Exp Med (2011) 208(8):1621–34 10.1084/jem.20102110 21788409 PMC3149221

[69] Sanjabi S Mosaheb MM Flavell RA Opposing effects of TGF-beta and IL-15 cytokines control the number of short-lived effector CD8+ T cells. Immunity (2009) 31(1):131–44 10.1016/j.immuni.2009.04.020 19604492 PMC2765785

[70] Hu Y Cauley L Antigen and transforming growth factor Beta receptors contribute to long term functional and phenotypic heterogeneity of memory CD8 T cells. Front Immunol (2013) 4:227 10.3389/fimmu.2013.00227 23964275 PMC3740294

[71] Sheridan BS Lefrancois L Regional and mucosal memory T cells. Nat Immunol (2011) 12(6):485–91 10.1038/ni.2029 21739671 PMC3224372

[72] Carlson CM Turpin EA Moser LA O’Brien KB Cline TD Jones JC Transforming growth factor-beta: activation by neuraminidase and role in highly pathogenic H5N1 influenza pathogenesis. PLoS Pathog (2010) 6(10):e1001136 10.1371/journal.ppat.1001136 20949074 PMC2951376

[73] Schultz-Cherry S Hinshaw VS Influenza virus neuraminidase activates latent transforming growth factor beta. J Virol (1996) 70(12):8624–9 8970987 10.1128/jvi.70.12.8624-8629.1996 PMC190955

[74] Wilson MS Wynn TA Pulmonary fibrosis: pathogenesis, etiology and regulation. Mucosal Immunol (2009) 2(2):103–21 10.1038/mi.2008.85 19129758 PMC2675823

[75] Ely KH Cookenham T Roberts AD Woodland DL Memory T cell populations in the lung airways are maintained by continual recruitment. J Immunol (2006) 176(1):537–43 10.4049/jimmunol.176.1.537 16365448

[76] Wakim LM Gupta N Mintern JD Villadangos JA Enhanced survival of lung tissue-resident memory CD8(+) T cells during infection with influenza virus due to selective expression of IFITM3. Nat Immunol (2013) 14(3):238–45 10.1038/ni.2525 23354485

[77] Teijaro JR Turner D Pham Q Wherry EJ Lefrancois L Farber DL Cutting edge: tissue-retentive lung memory CD4 T cells mediate optimal protection to respiratory virus infection. J Immunol (2011) 187(11):5510–4 10.4049/jimmunol.1102243 22058417 PMC3221837

[78] Turner DL Bickham KL Thome JJ Kim CY D’Ovidio F Wherry EJ Lung niches for the generation and maintenance of tissue-resident memory T cells. Mucosal Immunol (2014) 7(3):501–10 10.1038/mi.2013.67 24064670 PMC3965651

[79] Belkin VM Belkin AM Koteliansky VE Human smooth muscle VLA-1 integrin: purification, substrate specificity, localization in aorta, and expression during development. J Cell Biol (1990) 111(5 Pt 1):2159–70 10.1083/jcb.111.5.2159 2229189 PMC2116325

[80] Hemler ME VLA proteins in the integrin family: structures, functions, and their role on leukocytes. Annu Rev Immunol (1990) 8:365–400 10.1146/annurev.iy.08.040190.002053 2188667

[81] Miner JH Sanes JR Collagen IV alpha 3, alpha 4, and alpha 5 chains in rodent basal laminae: sequence, distribution, association with laminins, and developmental switches. J Cell Biol (1994) 127(3):879–91 10.1083/jcb.127.3.879 7962065 PMC2120241

[82] Sado Y Kagawa M Naito I Ueki Y Seki T Momota R Organization and expression of basement membrane collagen IV genes and their roles in human disorders. J Biochem (1998) 123(5):767–76 10.1093/oxfordjournals.jbchem.a022003 9562604

[83] Wright JR Immunoregulatory functions of surfactant proteins. Nat Rev Immunol (2005) 5(1):58–68 10.1038/nri1528 15630429

[84] Qaseem AS Sonar S Mahajan L Madan T Sorensen GL Shamji MH Linking surfactant protein SP-D and IL-13: implications in asthma and allergy. Mol Immunol (2013) 54(1):98–107 10.1016/j.molimm.2012.10.039 23220073

[85] Zaid A Mackay LK Rahimpour A Braun A Veldhoen M Carbone FR Persistence of skin-resident memory T cells within an epidermal niche. Proc Natl Acad Sci U S A (2014) 111(14):5307–12 10.1073/pnas.1322292111 24706879 PMC3986170

[86] Verbist KC Klonowski KD Functions of IL-15 in anti-viral immunity: multiplicity and variety. Cytokine (2012) 59(3):467–78 10.1016/j.cyto.2012.05.020 22704694 PMC3422395

[87] Shen CH Ge Q Talay O Eisen HN Garcia-Sastre A Chen J Loss of IL-7R and IL-15R expression is associated with disappearance of memory T cells in respiratory tract following influenza infection. J Immunol (2008) 180(1):171–8 10.4049/jimmunol.180.1.171 18097017 PMC2709277

[88] Kohlmeier JE Miller SC Woodland DL Cutting edge: antigen is not required for the activation and maintenance of virus-specific memory CD8+ T cells in the lung airways. J Immunol (2007) 178(8):4721–5 10.4049/jimmunol.178.8.4721 17404250

[89] Taylor BC Zaph C Troy AE Du Y Guild KJ Comeau MR TSLP regulates intestinal immunity and inflammation in mouse models of helminth infection and colitis. J Exp Med (2009) 206(3):655–67 10.1084/jem.20081499 19273626 PMC2699121

[90] Masopust D Vezys V Wherry EJ Barber DL Ahmed R Cutting edge: gut microenvironment promotes differentiation of a unique memory CD8 T cell population. J Immunol (2006) 176(4):2079–83 10.4049/jimmunol.176.4.2079 16455963

[91] Schenkel JM Fraser KA Masopust D Cutting edge: resident memory CD8 T cells occupy frontline niches in secondary lymphoid organs. J Immunol (2014) 192(7):2961–4 10.4049/jimmunol.1400003 24600038 PMC3965619

[92] Zammit DJ Turner DL Klonowski KD Lefrancois L Cauley LS Residual antigen presentation after influenza virus infection affects CD8 T cell activation and migration. Immunity (2006) 24(4):439–49 10.1016/j.immuni.2006.01.015 16618602 PMC2861289

[93] Kim TS Hufford MM Sun J Fu YX Braciale TJ Antigen persistence and the control of local T cell memory by migrant respiratory dendritic cells after acute virus infection. J Exp Med (2010) 207(6):1161–72 10.1084/jem.20092017 20513748 PMC2882836

[94] Moyron-Quiroz JE Rangel-Moreno J Kusser K Hartson L Sprague F Goodrich S Role of inducible bronchus associated lymphoid tissue (iBALT) in respiratory immunity. Nat Med (2004) 10(9):927–34 10.1038/nm1091 15311275

[95] de Bree GJ van Leeuwen EM Out TA Jansen HM Jonkers RE van Lier RA Selective accumulation of differentiated CD8+ T cells specific for respiratory viruses in the human lung. J Exp Med (2005) 202(10):1433–42 10.1084/jem.20051365 16301748 PMC2212987

[96] Piet B de Bree GJ Smids-Dierdorp BS van der Loos CM Remmerswaal EB von der Thusen JH CD8(+) T cells with an intraepithelial phenotype upregulate cytotoxic function upon influenza infection in human lung. J Clin Invest (2011) 121(6):2254–63 10.1172/JCI44675 21537083 PMC3104744

[97] Purwar R Campbell J Murphy G Richards WG Clark RA Kupper TS Resident memory T cells (T(RM)) are abundant in human lung: diversity, function, and antigen specificity. PLoS One (2011) 6(1):e16245 10.1371/journal.pone.0016245 21298112 PMC3027667

[98] Selin LK Cornberg M Brehm MA Kim SK Calcagno C Ghersi D CD8 memory T cells: cross-reactivity and heterologous immunity. Semin Immunol (2004) 16(5):335–47 10.1016/j.smim.2004.08.014 15528078 PMC7128110

